# Assessment of TNF-α inhibitors in airway involvement of relapsing polychondritis: A systematic review

**DOI:** 10.1097/MD.0000000000017768

**Published:** 2019-11-01

**Authors:** Josette Biya, Sandra Dury, Jeanne-Marie Perotin, Claire Launois, Maxime Dewolf, Gaëtan Deslée, François Lebargy

**Affiliations:** a Department of Respiratory Diseases, Reims University Hospital; b EA 4683 Medical and Pharmacological Sciences; c UMRS 903, Reims University Hospital, Reims, France.

**Keywords:** airways, relapsing polychondritis, TNF-α inhibitors

## Abstract

Relapsing polychondritis (RP) is a rare immune-mediated disease affecting cartilaginous structures. Respiratory tract manifestations are frequent and constitute a major cause of morbidity and mortality. The present review of the literature was designed to assess the efficacy of tumor necrosis factor alpha (TNF-α) inhibitors in respiratory tract involvement of RP.

A MEDLINE literature search was performed from January 2000 to December 2016 to identify all studies and case reports of anti-TNF-α therapy in RP. Articles published in English or French concerning patients with respiratory tract involvement were eligible. Two authors (JB, FL) independently reviewed and extracted data concerning each patient and 2 personal cases were added. Treatment efficacy was assessed according to systemic and/or respiratory criteria.

A total of 28 patients (mean age: 41.6 years; 16 females/12 males) were included in the final analysis. Anti-TNF-α therapy was associated with improved health status and respiratory symptoms in 67.8% and 60.1% of cases, respectively.

These results suggest that TNF-α inhibitors could be considered for the treatment of respiratory tract involvement of RP.

## 1 Introduction

Relapsing polychondritis (RP) is a rare immune-mediated disease affecting the cartilage of the nose, ears, tracheobronchial tree, peripheral joints, and proteoglycan-rich structures such as the inner ear, eyes, and cardiovascular system, mainly occurring during the 5th decade, with an equal sex ratio.^[1–5]^ The diagnosis of RP is based on the criteria established by McAdam in 1976, and modified in 1979 by Damiani and Levine^[1,2]^ (Table 1). The pathophysiology of RP is unclear, but potentially involves type II-collagen and matrilin-1.^[4]^ The target antigens remain unknown.

**Table 1:**
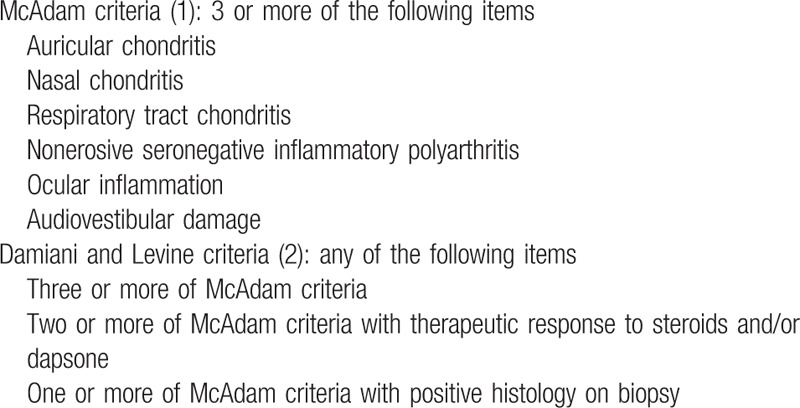
Diagnostic criteria for relapsing polychondritis.

Respiratory manifestations occur in up to 50% of patients with RP and are a major source of morbidity and mortality.^[5–8]^ Systemic corticosteroid therapy is considered to be the cornerstone of treatment. Immunosuppressive agents, such as methotrexate, azathioprine, cyclophosphamide, ciclosporin, and mycophenolate mofetil, are used as corticosteroid-sparing agents, or in the case of severe organ involvement.^[3,9]^ Biological agents, especially tumor necrosis factor alpha (TNF-α) inhibitors, have been considered as new treatment options. However, the exact impact of these treatments on airway involvement in RP has not been clearly determined. The present review of the literature was designed to assess the efficacy of TNF-α inhibitors in respiratory tract involvement of RP.

## 2 Methods

### 2.1 Literature search

A MEDLINE search of the English and French literature was performed to identify studies and cases reporting RP respiratory tract involvement and the use of TNF-α inhibitors. This search covered the period from the first use of anti-TNF-α therapy in January 2000 to December 2016. The following search terms were used: “Relapsing polychondritis,” “TNF-alpha blockers,” “TNF-alpha antagonists,” “TNF-alpha inhibitors” “anti-TNF-alpha,” “infliximab,” “etanercept,” “adalimumab,” “golimumab,” and “certolizumab.” We also searched for additional articles from the reference list of the relevant articles selected.

### 2.2 Inclusion criteria

Cases were selected when they met all of the following 3 criteria: Diagnosis of RP based on the criteria proposed by McAdam or Damiani and Levine (Table 1); Respiratory tract involvement reported as clinical symptoms (cough, dyspnea, hoarseness, stridor, bronchospasm, and laryngotracheal tenderness), and/or obstructive defect on pulmonary function tests and/or tracheal or bronchial involvement (narrowing, thickening, stenosis or calcifications of the tracheal, and/or bronchial wall) on computed tomography (CT) scan and/or abnormal endoscopic findings (tracheomalacia, stenosis, and inflammation); and Use of 1 or more TNF-α inhibitors during the course of the disease. Articles were excluded when specific data could not be extracted. References were reviewed by 2 independent investigators (JB and FL). Decision for inclusion was based on a consensus involving a 3rd investigator (SD). Two previously unpublished personal cases were also added. In accordance with the Jardé law in France, access to patient data was approved by the French national commission for personal data protection (CNIL, Comité National de l’Information et des Libertés) (no 2049775 v 0) and informed consent for inclusion was waived because of the retrospective noninterventional design of this study and anonymous management of the patients’ data.

### 2.3 Assessment criteria

Systemic response was defined by improvement of overall health status and/or clinical improvement of chondritis, and/or steroid sparing or withdrawal.

Respiratory response was defined as improvement of respiratory symptoms and/or pulmonary function tests and/or radiological findings and/or endoscopic features. Adverse events related to anti-TNF-α therapy were also reported.

## 3 Results

**Figure 1:**
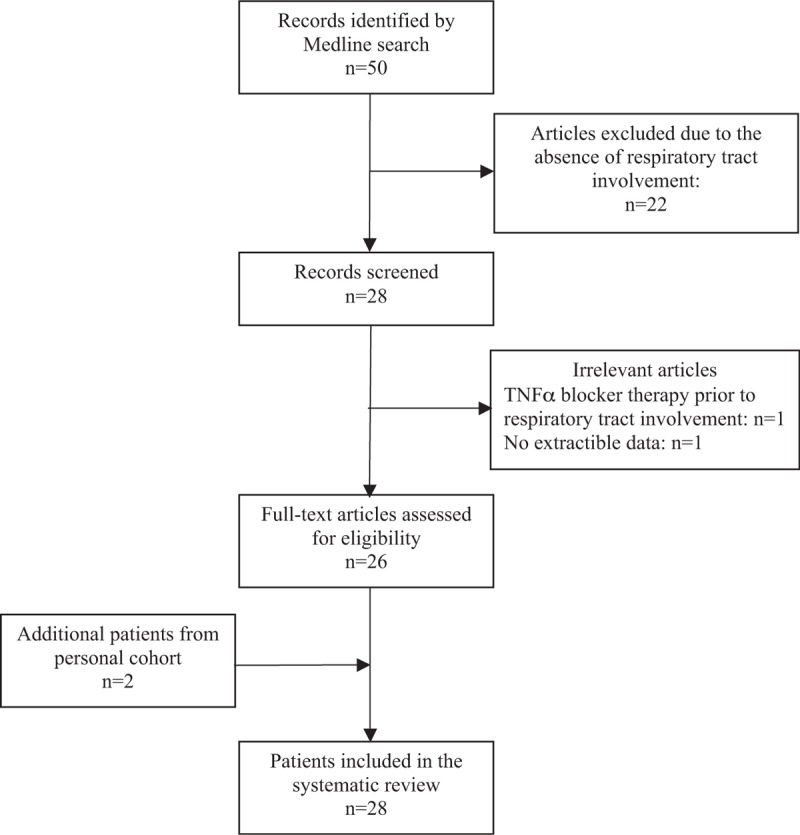
Patient selection flow chart.

Figure 1 shows the flowchart of the selected articles.^[11–35]^ The final analysis was carried out on a total of 28 patients, including 2 unpublished personal cases.

### 3.1 Demographic and respiratory features

Patient characteristics are presented in Table 2. Mean age at diagnosis was 41.6 years (6–69). Sixteen patients (57.1%) were women. Other concomitant autoimmune diseases were reported in 5 cases (17.8%). The most common manifestations were laryngeal symptoms (n = 21, 75%) (hoarseness n = 11, stridor n = 2, dysphonia n = 1, laryngeal tenderness n = 2, or throat pain n = 2, unspecified “laryngeal symptoms” n = 4) and respiratory symptoms (n = 21, 75%) (cough n = 6, dyspnea n = 13, tracheal tenderness n = 2, bronchospasm n = 1, unspecified “tracheal symptoms” n = 2). Acute respiratory failure was reported in 6 patients (21.4%). Six of the 9 patients for whom pulmonary function tests were available exhibited an obstructive pattern. Seven of the 13 patients investigated by CT scan presented tracheal thickening. Fiberoptic bronchoscopy was performed in 8 patients and revealed tracheomalacia (n = 4), tracheal inflammation (n = 3), or laryngeal inflammation and subglottic stenosis (n = 2).

**Table 2:**
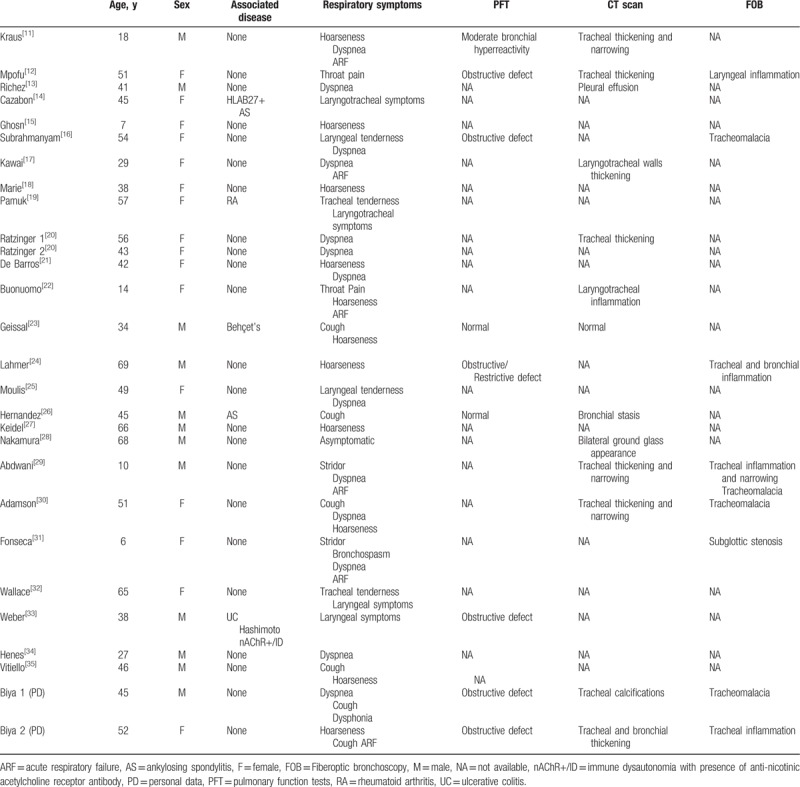
Demographic and clinical data.

### 3.2 Drug regimen

All patients had previously received immunosuppressive agents, including steroids (100%), methotrexate (70%), cyclophosphamide (41%), azathioprine (22%), and rarely ciclosporin, mycophenolate mofetil, immunoglobulins, hydrochloroquine, tacrolimus, or rituximab (data not shown).

Infliximab, etanercept, and adalimumab were used in 75% (n = 21), 28.6% (n = 8), and 21.4% (n = 6) of cases, respectively (Table 3). Six patients were treated with more than 1 TNF-α inhibitor due to adverse effects or lack of efficacy (11, 16, 25, 27, 31, Biya 1). Infliximab was administered at doses of 3 to 10 mg/kg/infusion, etanercept 25 mg was administered twice weekly by subcutaneous injection, and adalimumab 40 mg was administered once a fortnight. A TNF-α inhibitor was associated with other immunosuppressive agents, including steroids (100%), methotrexate (50%), cyclophosphamide (8.3%), azathioprine (4.2%), sulfasalazine (4.2%), or dapsone (4.2%) (data available for 24 patients) (Table 3).

**Table 3:**
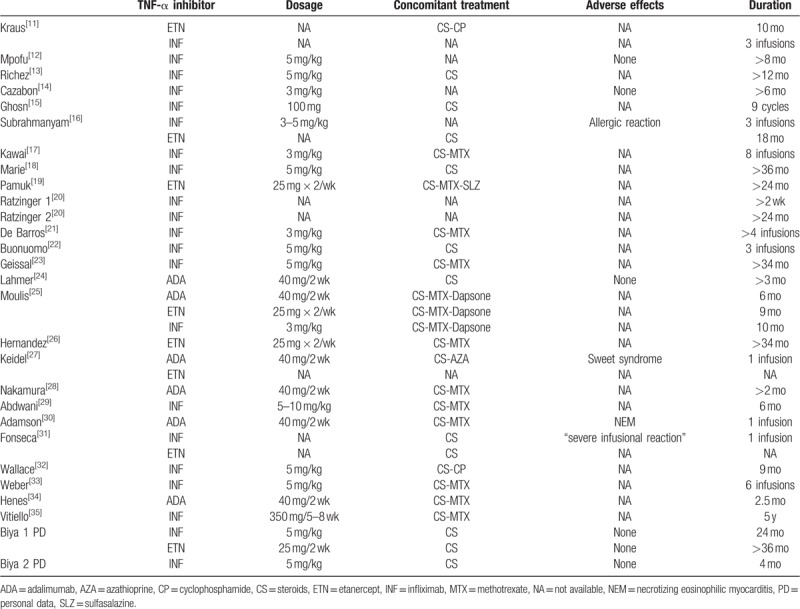
Drug regimen.

Treatment duration reached 5 years for infliximab,^[35]^ 36 months for etanercept (Biya 1), and 6 months for adalimumab.^[25]^ Three patients received only 1 injection of anti-TNF-α inhibitor due to adverse effects.^[27,30,31]^

### 3.3 Adverse effects of TNF-α inhibitors

Adverse effects are reported in Table 4. In 2 cases, infliximab caused a systemic or local hypersensitivity reaction leading to a switch from infliximab to etanercept in both cases.^[16,31]^ Infliximab induced an allergic chest reaction in 1 case with no impact on treatment.^[16]^ Adalimumab therapy was complicated by an episode of Sweet syndrome requiring treatment discontinuation,^[27]^ and a case of fatal necrotizing eosinophilic myocarditis.^[30]^

**Table 4:**
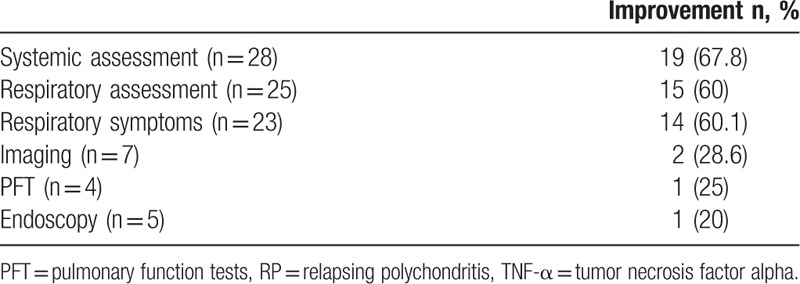
Assessment and efficacy of TNF-α inhibitors in RP.

### 3.4 Assessment of the efficacy of anti-TNF-α therapy

Assessment of systemic manifestations after anti-TNF-α therapy was reported in all cases (Table 4). Nineteen of the 28 cases (67.8%) showed improvement of general health status (11–16, 18–21, 23–24, 26–28, 31, 35, Biya 1). Improvement of chondritis was reported in 13 out of 19 patients (68.4%) (13–16, 18–19, 21, 24, 26, 28–29, 35, Biya 1).

Respiratory assessment after anti-TNF-α therapy was available for only 25 patients (11–18, 20, 22, 24–35, Biya 1, Biya 2). Fifteen of these patients were improved (60%), but this assessment was only based on respiratory symptoms in 14 patients. Therefore, only 2 patients displayed respiratory improvement in terms of both symptoms and chest imaging and/or endoscopic features and/or PFT.^[11,12]^

Fourteen patients were assessed for both ear/nose chondritis and respiratory tract. Among them, 12 patients had a similar response with an improvement in 7 patients and worsening in 5 patients.

## 4 Discussion

Respiratory tract involvement in RP occurs in up to 50% of patients during the course of the disease and is a leading cause of death due to airway collapse/narrowing and airway infections.^[5–7]^ Although epidemiological data suggest an equal gender distribution of RP, respiratory symptoms seem to be more frequent in women.^[5]^ Hoarseness and laryngeal tenderness over the thyroid cartilage and anterior trachea are the most common symptoms. Other symptoms include dry cough, dyspnea, inspiratory stridor, wheezing, and more rarely, hemoptysis.^[36,37]^ Airway stenosis is common and associated with airflow obstruction, as reported by Tillie-Leblond et al in 8 out of 9 patients with RP associated with respiratory symptoms.^[38]^ Assessment of expiratory and inspiratory flow-volume curves and airway resistance may be useful to identify the site and severity of the obstructive process.^[39]^ The characteristic findings on CT scan are thickening of tracheal and/or bronchial walls with sparing of posterior membrane, calcifications involving airway walls, and focal or diffuse airway stenosis.^[36,38,40]^ Dynamic expiratory CT scans demonstrate dynamic abnormalities in more than 90% of patients, including tracheomalacia and/or air trapping, although only half of them present inspiratory abnormalities.^[40]^ Bronchoscopy demonstrates abnormalities of the tracheobronchial tree in most patients, corresponding to mucosal inflammation, possibly associated with tracheal stenosis or total collapse of the trachea.^[36]^ Although well tolerated, bronchoscopy must be performed carefully due to the risk of severe airway collapse.^[38]^

Few data are available concerning the associations between airway involvement and RP activity. Pathology findings suggest that the initial phase of the disease is characterized by inflammation of the tracheal wall with cartilage involvement leading to thickening and focal or diffuse airway stenosis. As the disease progresses, disruptions in the cartilage structure and fibrosis replacement lead to flabbiness and collapse of the airways.^[5]^ It is difficult to distinguish between respiratory symptoms related to acute flare of the disease and those related to airway destruction. The Relapsing Polychondritis Disease Activity Index (RPDAI) study group recently proposed a rating scale using 27 weighted items in order to provide objective means of assessment of disease activity.^[41]^ High weighted scores have been attributed to bronchial chondritis, but with no distinction between active inflammatory flares and irreversible destructive airway damage. Assessment of RP activity using fluorodeoxyglucose positron emission tomography (FDG/PET) could be a promising approach. In a retrospective study of 13 RP patients assessed by FDG/PET, 9 patients presented increased FDG uptake in the tracheobronchial tree, associated with CT abnormalities in every case.^[42]^ Decreased FDG uptake in upper and lower airways was also observed in 5 patients after treatment.^[43]^ However, further studies are needed to determine the sensitivity and specificity of FDG/PET to assess airway involvement in RP.

A standardized treatment protocol has not been established due to the rarity of the disease. Current therapy is largely empiric and based on case reports. Steroids remain the mainstay during disease flares and as maintenance therapy at lower doses to prevent relapses. Severe forms of RP may require intravenous pulses of high-dose steroids or cyclophosphamide in addition to steroids. Immunosuppressants, such as methotrexate, azathioprine, dapsone, ciclosporin, mycophenolate, or intravenous immunoglobulin, have been proposed as steroid-sparing agents, but their efficacy has yet to be established. TNF-α inhibitors have been tested in many connective tissue diseases and have been shown to be effective in rheumatoid arthritis and ankylosing spondylitis, but few data are available in RP. In a recent systematic review of the literature comprising case reports and small series, infliximab was effective in 14 of 31 treated patients (45%), but ineffective in 13 patients (42%).^[10]^ A few patients were treated with etanercept (n = 9) or adalimumab (n = 4), successfully in 55% and 50% of cases, respectively.^[10]^ To our knowledge, this is the first study to assess the efficacy of TNF-α inhibitors on RP airway involvement. The results of this study demonstrate that TNF-α inhibitors are effective on systemic manifestations and respiratory involvement in 67.8% and 60.1% of cases, respectively. However, efficacy endpoints of biologics are poorly defined and differ from 1 case report to another. Of note in most cases, the efficacy of TNF-α inhibitors was assessed only on subjective respiratory symptoms. Indeed, some of them such as dyspnea, wheezing, or stridor cannot be used to distinguish laryngeal and tracheal involvement.

This study presents several limitations. Firstly, many data were missing for the cases selected in this systematic review. In 3 out of 28 cases, no information was available on respiratory follow-up after anti-TNF-α therapy. Furthermore, only 7 of the 13 patients investigated before treatment by chest CT scan were reassessed after treatment. Only 2 patients were assessed by FDG/PET. Interestingly, our patient did not show any tracheal ^18^FDG uptake on FDG/PET, but was improved by TNF-α inhibitors, with a reduction of symptoms, exacerbations, and hospitalizations. In the absence of objective criteria, response to treatment should therefore be interpreted with caution. Secondly, respiratory manifestations reported in 3 cases do not correspond to the specific airway disease usually related to RP. One patient had pleural effusion secondary to myocarditis,^[13]^ another had chronic obstructive pulmonary disease,^[24]^ and the last patient had a nonspecific ground glass appearance on CT scan.^[28]^ The efficacy of TNF-α inhibitors may therefore be overestimated in this systematic review. Thirdly, many patients received concomitant treatments that could interfere with TNF-α inhibitors. Airway investigations were rarely performed and were incomplete, preventing inflammatory lesions to be distinguished from irreversible airway damage. Systematic assessment of airway lesions in RP, including chest CT scan, pulmonary function test, and endoscopic investigation, would be useful.

## 5 Conclusion

Respiratory tract involvement is common in RP. Because of the rarity of this disease, no standardized treatment protocol is currently available. This review of the literature suggests the efficacy of TNF-α inhibitors on systemic and respiratory symptoms. However, this efficacy needs to be confirmed by more detailed case reports of respiratory involvement.

## Author contributions

**Conceptualization:** Josette Biya, Sandra Dury, Jeanne-Marie Perotin, Maxime Dewolf, Claire Launois, Gaëtan Deslée, Francois Lebargy.

**Data curation:** Josette Biya, Sandra Dury, Francois Lebargy.

**Formal analysis:** Josette Biya, Sandra Dury, Francois lebargy.

**Methodology:** Josette Biya, Sandra Dury, Francois Lebargy.

**Project administration:** Josette Biya, Sandra Dury, Francois Lebargy.

**Supervision:** Josette Biya, Sandra Dury, Gaëtan Deslée, Francois Lebargy.

**Validation:** Josette Biya, Sandra Dury, Francois Lebargy.

**Visualization:** Josette Biya, Sandra Dury, Jeanne-Marie Perotin, Maxime Dewolf, Claire Launois, Gaëtan Deslée, Francois Lebargy.

**Writing – original draft:** Josette Biya, Sandra Dury, Jeanne-Marie Perotin, Maxime Dewolf, Claire Launois, Gaëtan Deslée, Francois Lebargy.

**Writing – review & editing:** Josette Biya, Sandra Dury, Francois Lebargy.
